# Flavin Adenine Dinucleotide-Related Adverse Event Necessitating Cardiopulmonary Resuscitation in an Infant

**DOI:** 10.7759/cureus.22143

**Published:** 2022-02-11

**Authors:** Shunsuke Fujii, Kenichi Tetsuhara, Suzu Imamura, Mamoru Muraoka, Sayo Suzuki

**Affiliations:** 1 Department of Critical Care Medicine, Fukuoka Children’s Hospital, Fukuoka, JPN; 2 Department of Pediatric Cardiology, Fukuoka Children’s Hospital, Fukuoka, JPN

**Keywords:** vitamin b2, mitochondrial disease, flavin adenine dinucleotide, cardiopulmonary resuscitation, adverse event

## Abstract

Mitochondrial rescue drugs, such as vitamin B2, are a treatment modality which can be considered in unexplained cases of cardiac arrest or impaired consciousness in which mitochondrial diseases are considered in the differential. Although case reports exist of children developing a drop in their blood pressure following administration of intravenous flavin adenine dinucleotide (FAD) sodium as vitamin B2, we present the first reported case of a child requiring cardiopulmonary resuscitation (CPR) following FAD sodium infusion for severe bradycardia and hypotension. Intravenous infusion of FAD sodium should be administered as slowly as possible, with careful monitoring of blood pressure and heart rate.

## Introduction

Mitochondrial rescue drugs, such as vitamin B2, may be administered for suspected metabolic diseases due to cardiac arrest, impaired consciousness, or lactic acidosis with no apparent cause [1-4]. Flavin adenine dinucleotide (FAD) sodium is commonly used in vitamin B2 preparations. There have been reports of adverse events with the use of FAD sodium, such as hypotension, bradycardia, chest discomfort, and neutropenia, but there have been no reports of cardiopulmonary resuscitation (CPR) being required [1,5,6]. We report the first case of an infant who underwent CPR for severe bradycardia and hypotension due to intravenous infusion of FAD sodium.

## Case presentation

The patient, a seven-month-old girl weighing 3.8 kg, had undergone pulmonary artery banding for atrioventricular septal defect and also had other congenital malformations such as hip dislocation and imperforate anus. She was hospitalized during the initial visit with a fever and cough and primary diagnosis of acute bronchitis. Five hours after admission, she suffered sudden cardiac arrest and CPR was performed. Although her heart resumed beating unassisted after one hour of CPR, her cardiac function was markedly impaired (left ventricular ejection fraction of 30% on echocardiography). Therefore, she was transferred to a facility that could immediately provide extracorporeal membrane oxygenation (ECMO). After return of spontaneous circulation, arterial blood gas analysis revealed a metabolic acidosis (pH: 7.301, partial pressure of arterial carbon dioxide [PaCO_2_]: 22.6 mmHg, bicarbonate: 13.5 mmol/L, base deficit: -13.9 mmol/L, lactate: 99 mg/dL) and hyperammonemia (210 µg/dL). Because the cardiac arrest had no obvious cause, the differential diagnosis included metabolic diseases (e.g., mitochondrial diseases); therefore, drugs for mitochondrial diseases, riboflavin sodium phosphate as vitamin B2 (25 mg/kg over 15 minutes twice a day), fursultiamine hydrochloride, pyridoxal phosphate hydrate, ascorbic acid, tocopherol acetate, biotin, ubidecarenone, and levocarnitine, were administered with treatment for heart failure. Her cardiac function gradually recovered without ECMO, and left ventricular ejection fraction improved from 30% to 50% on echocardiography. She was transferred back to our hospital on day seven. At the time of readmission, she had a respiratory rate of 35 breaths/min; oxygen saturation of 92% (fraction of inspired oxygen of 40% on mechanical ventilation); blood pressure of 115/55 mmHg; heart rate of 130 beats per minute (bpm) (dobutamine: 3 µg/kg/min, milrinone: 0.3 µg/kg/min); and axillary temperature of 36.5°C. The treatment at the previous hospital was continued, except that FAD sodium was substituted for vitamin B2, because riboflavin was not adopted by our hospital. FAD sodium, 25 mg/kg, was scheduled to be administered over one hour twice a day.

On day eight, during continuous intravenous infusion of FAD sodium (25 mg/kg/h), her systolic blood pressure gradually dropped to approximately 60 mmHg. We decided to administer a fluid challenge to assess fluid responsiveness. Immediately after slow bolus intravenous infusion of the remaining 30 mg FAD sodium, her systolic blood pressure dropped to approximately 30 mmHg with severe sinus bradycardia (50 bpm). CPR was performed for 30 seconds due to severe bradycardia, and her blood pressure and heart rate improved immediately. The drug information for FAD sodium did not refer to hypotension, bradycardia, or cardiac arrest as adverse reactions; because her blood pressure and heart rate remained unchanged during the administration of riboflavin sodium phosphate, we assumed that the administration of FAD sodium was not attributed to bradycardia and hypotension. FAD sodium was intravenously infused again as per schedule via a slow bolus. Then, transient bradycardia (from 120 bpm to 70 bpm) and hypotension (from 112/67 mmHg to 60/27 mmHg) recurred, which improved within two minutes of discontinuation of FAD sodium infusion. We discontinued FAD sodium as the FAD sodium reproducibly affected the blood pressure and heart rate. All remaining metabolic drugs were discontinued on day 14 because the screening results were negative for metabolic disease and her clinical course had improved. Her general condition remained stable after the discontinuation of metabolic drugs.

## Discussion

FAD sodium, formed by the binding of a riboflavin moiety (vitamin B2) to a phosphatase group of an adenosine diphosphate molecule [7], is commonly used in vitamin B2 preparations. Additionally, vitamin B2 is a component of electron transfer complex II, which is used as a therapeutic agent in some mitochondrial diseases [1-4]. Mitochondrial rescue should be considered as early as possible to avoid delaying appropriate treatment in cases of lactic acidosis, hypoglycemia, hyperammonemia, and when mitochondrial disease is suspected, even before a definite diagnosis is made [2,3]. Mitochondrial rescue is assumed not to cause any lethal side effects such as severe bradycardia or hypotension [3].

Hypotension during intravenous infusion of FAD sodium has been described in several children, but as far as we know, no other children have required CPR as a result [5,6]. Animal experiments suggest that the activation of nitric oxide synthase in cardiac pacemaker and muscle cells and a pronounced vasodilator response causes hypotension and bradycardia induced by intravenous FAD sodium [4]. According to reports of FAD sodium administration and hypotension, FAD sodium is administered at an average rate of 2.1 mg/kg/h and 7.2 mg/kg/h in patients without and with hypotension, respectively [1]. In this case, FAD sodium was administered at a rate of 25 mg/kg/h, and in addition, as slow bolus. The causal relationship between two events is evaluated in terms of "strength," "consistency," "specificity," "temporality," "biological gradient," "plausibility," "coherence," "experiment," and "analogy" [8]. In this case, there was reproducibility in bradycardia and hypotension immediately after FAD sodium administration ("consistency" and "temporality"), and the symptoms were more intense with slow bolus infusion than those with continuous intravenous infusion ("biological gradient"). In addition to the previous literature ("strength," "specificity," "plausibility," "coherence," and "experiment") [1,4], this case supports the idea that bradycardia and hypotension are adverse events of FAD sodium. Furthermore, the fact that she developed severe bradycardia and hypotension requiring CPR may be related to the presence of underlying cardiac disease.

## Conclusions

Intravenous infusion of FAD sodium should be administered as slowly as possible, with careful monitoring of blood pressure and heart rate. FAD sodium is used as a mitochondrial rescue drug. It can cause bradycardia and hypotension to the point that CPR is required. Therefore, intravenous infusion of FAD sodium should be administered as slowly as possible, with careful monitoring of blood pressure and heart rate.
